# Health professionals’ perceptions of planned home birth care within the Brazilian health system

**DOI:** 10.1186/s12884-023-06161-9

**Published:** 2023-12-08

**Authors:** Jannaina Campos Beviláqua, Laena Costa dos Reis, Valdecyr Herdy Alves, Lucia Helena Garcia Penna, Silvio Éder Dias da Silva, Andressa Tavares Parente, Fabianne de Jesus Dias Sousa, Bianca Dargam Gomes Vieira, Audrey Vidal Pereira, Maura Eduarda Sousa Fernandes, Diego Pereira Rodrigues

**Affiliations:** 1 School of Nursing, Federal University of Pará, Belém, Pará Brazil; 2 State Department of Health of the State of Pará, Belém, Pará Brazil; 3 School of Nursing, Federal Fluminense University, Niterói, Rio de Janeiro Brazil; 4 School of Nursing, State University of Rio de Janeiro, Rio de Janeiro, Rio de Janeiro Brazil

**Keywords:** Obstetric nursing, Maternal and child health, Humanized childbirth, Humanization of care, Home childbirth

## Abstract

**Background:**

The American College of Obstetricians and Gynecologists, in its opinion of the Committee on Midwifery Practice, points out that planned home birth is a woman's and family's right to experience, but also to choose and be informed about, their baby's place of birth. The aim of this study was to understand obstetric nurses' perceptions of planned home childbirth care within the framework of the Brazilian obstetric model.

**Method:**

A qualitative study, with Snowball Sampling recruitment, totaling 20 obstetric nurses through semi-structured interviews between September 2022 and January 2023, remotely, using the Google Meet application and the recording feature. After the data had been collected, the material was transcribed in full and subjected to content analysis in the thematic modality with the support of ATLAS.ti 8.0 software.

**Results:**

Obstetric care at home emerged as a counterpoint to hospital care and the biomedical model, providing care at home based on scientific evidence and humanization, bringing qualified information as a facilitator of access and financial costs as an obstacle to effective home birth.

**Conclusion:**

Understanding obstetric nurses' perceptions of planned home birth care in the context of the Brazilian obstetric model shows the need for progress as a public policy and for strategies to ensure quality and regulation**.**

## Background

The American College of Obstetricians and Gynecologists (ACOG), in its Committee on the Practice of Obstetrics, points out that planned home birth (PHB) is a right for women and their families not only to experience it but also to choose and be informed about their baby's place of childbirth [1].

Women are increasingly seeking alternative models that break with access to traditional services due to established institutional norms; a more vertical relationship between the woman/family and the health professional; a lack of acceptance and empathy; care centered on the health professional and their practice, not based on scientific evidence; as well as disrespect, discrimination, neglect, and violence in the field of reproductive health [2–6].

Countries such as the United Kingdom, the Netherlands, and Australia, as well as Nordic countries like Sweden, Denmark, Finland, Iceland, and Norway, guarantee respect for the choice and rights of women and their families for PHB, where there is an association of greater safety and fewer perinatal risks, both in terms of the rate of transfers to hospital units and mortality [7].

The main reason for the disagreement of many health professionals, especially doctors, is the safety of childbirth. A recent study demonstrated PHB regardless of place of childbirth, with a high vaginal delivery rate of 92%, as well as safety and positive maternal and neonatal outcomes, indicating that even in cases where transfer to hospital is necessary, PHB is still associated with uncomplicated childbirths [8]. In places where there is more integration between providers and health services, the perinatal and neonatal mortality rate was like hospital childbirths [9].

A study, based on a systematic review with meta-analysis on maternal and neonatal morbidity after home birth compared to planned hospital childbirth with women with normal risk pregnancies, indicated that vaginal childbirth was significantly higher in the PDP group [10]. Women in the home birth group were less likely to undergo cesarean section, as well as less likely to receive medical interventions, than the hospital group. The risk of postpartum hemorrhage was also lower in the PHB group than in the other group, and the two groups were similar in relation to neonatal morbidity and mortality [10]. These data corroborate the scientific literature, which shows that PHB is a method of choice for women and their families that has increased the number of women seeking PHB [2–4] and guarantees safety [7–11].

Childbirth and delivery in Brazil are still a hospital-centered model, centered on health professionals. PHB, on the other hand, is still not part of the reality for many Brazilian women, since most of them are small groups with greater purchasing power, a fact that is compounded by numerous obstacles, such as the lack of health professionals, integration between health services, investments in childbirth, support from medical entities and associations, and encouragement from both governmental organizations and the Brazilian government.

Home births in the country consist of small initiatives by teams of autonomous health professionals made up of obstetric nurses, obstetricians, and obstetricians, with obstetric nurses being the main providers of home birth care. These nurses are trained through professional specialization programs in obstetric nursing, with a minimum of 360 h, or in a residency program with a total of 5760 h. Of these, 80% are intended for practice lasting two years, with the obligation for the title to help with 20 prenatal cares, 20 deliveries, and 15 h of assistance to the newborn in the delivery room, in line with Resolutions 516/2016 and 672/2021 of the Federal Nursing Council [12].

The residency program differs in that it has a large practical workload and that professionals from the services mediate the training, complying with the proposals of the International Confederation of Midwives in relation to the professional competencies required for training [13, 14]. However, both types of training are not specific to home births but rather to work in obstetric care, especially throughout the pregnancy-puerperium cycle, which, with the experience acquired and their professional experience, enables these professionals to work with specialized PHB teams.

Regarding the rate of home childbirths in Brazil, it corresponds to less than 2.4% of births, with a predominance of childbirths in hospital units, such as maternity hospitals and childbirth centers [15]. The country has only one public home birth service, the Sofia Feldman Hospital, which is a benchmark for humanization in Brazil.

Thus, the Ministry of Health (MH) recognizes, through technical note no. 2/2021 [16] from the Department of Strategic Programmatic Actions of the Primary Health Care Secretariat, that the choice of place of childbirth should be centered on the role of the woman and ensure quality information on the risks and benefits of the different alternatives of place of childbirth. However, the note advises against PHB in the context of Brazilian obstetric care, which is not contained in its framework of procedures in the Unified Health System, translating PHB as a risk to the out-of-hospital environment and providing a lack of assistance to women who take this initiative, who will need high investment to guarantee their rights and expectations of their childbirth [2–5, 7–9].

Also, the Federal Nursing Council does not have any resolutions or regulations that guarantee support for the work of obstetric nurses in the PHB. The Federal Council of Medicine disagrees with their actions, and this discussion requires an opinion to resolve the problem of obstetric nurses providing care in the PHB.

Thus, home birth in Brazil requires a break with the hegemonic obstetric model, with the predominance of hospital childbirths, since home birth allows for greater bonding, a change in model, with a focus on humanization, respect for childbirth and women's rights [6, 11]. These women should receive informed care based on scientific evidence, with trained professionals, avoiding marginalization in the context of their choices. As well as the advancement of regulations and protocols to guarantee the legitimacy of professional practice in home birth care; remedying the difficulties with operational and infrastructure resources in care, as well as the shift towards investment, making it a focus of public health and the Brazilian Unified Health System, which would favor the universality and equity of PHB. This would strengthen obstetric nursing as a precursor of care for Brazilian women.

In this way, it is understood that the understanding of health professionals when they provide planned home birth care can help shape the meanings of the issue in society. Dialogues, research, and discussions about the PHB could allow greater access to this type of care for labor and childbirth, especially in the Brazilian obstetric model, and boost policies and measures to guarantee women's rights and prevent them from being annulled.

Given this scenario, the study's guiding question was: how do obstetric nurses who provide care for planned home births perceive PHB care in the reality of Brazilian women? In this way, the study aimed to understand obstetric nurses' perceptions of planned home birth care in the context of the Brazilian obstetric model.

## Methods

### Study design

This is a qualitative, descriptive, and exploratory study. Descriptive research is based on describing the characteristics of a particular population or phenomenon, while exploratory research aims to bring the researcher closer to the subject, to make them more accustomed to the facts and phenomena related to the research problem [17]. The study was based on the Consolidated Criteria for Reporting Qualitative Research (COREQ) tool, which is designed to help researchers describe the results of qualitative research with clarity and quality.

### Population, sample and data collection period

Twenty obstetric nurses who provide home birth care took part in the study, using the Snowball Sampling technique, also known as the snowball technique, for recruiting participants [18]. This strategy was used because it is a relatively small group of people who are in constant contact with each other, and as a productive way of building an exhaustive and representative sample. This sample of recruited interviewees will indicate the so-called "seed" people, resulting in new contacts based on a personal relationship, facilitating the interviewer's access based on reliability [18].

The first contact was made on the recommendation of the main researcher, who was aware of a professional involved in PHB care and met the following eligibility criteria: to be an obstetric nurse; to have worked in planned home births for at least a year. Obstetricians, doulas, and midwives were excluded. Subsequently, this participant referred the second participant who, in turn, referred the third and so on, until 20 interviews were completed, when theoretical saturation of the data was observed [19], as no new elements emerged to deepen the theorization of the object of study.

It should be emphasized that the obstetric nurses who took part in the study participated through their experiences with home birth, so they actively participated in the birth through their perceptions of home birth care in Brazil, with the challenges and rupture with the hegemonic Brazilian model. In this way, the nurses report their experiences of a place and belonging of their practice.

The data collection technique used was a semi-structured interview, with open and closed questions, portraying the identification profile of the professionals: gender; age; race/ethnicity/religion; marital status; length of experience in obstetric care and length of service in PHB and with the following research questions: How do you perceive home birth in the Brazilian context? How do you observe home birth care in Brazil? What is needed to advance planned home birth in the context of the guiding principles of the Brazilian health system?

The data was collected individually and remotely between September 2022 and January 2023, using the Google Meet application and the recording feature to capture the testimonies, with the prior authorization of the participants. The interviews were conducted in depth, with a single moment between the researcher and the interviewee, lasting an average of one hundred and ten minutes each, making it possible to investigate the perception of health professionals of planned home birth care.

### Data analysis

The statements were transcribed in full. After this stage, they were validated by the interviewees, who were then shown their testimonies via the WhatsApp messaging app, thus validating the data, and carrying out the treatment. It should be noted that data collection was only conducted by one researcher, with a doctoral degree, who mastered the technique used, thus avoiding a different approach, while the research team was able to act in the treatment and data analysis.

Thematic content analysis [20] was used to organize and process the data. First of all, a floating reading of each interview took place, with the observation of representative and relevant aspects, as described in the first stage, pre-analysis. In the second stage, the material was explored, followed by coding, relating the participants' speeches to the purpose of categorization [20].

At this stage, the functionality of ATLAS.ti 8.0 was used to code the excerpts from the testimonies, identifying the following meanings: humanization, obstetric model, scientific evidence, guidelines, and protocols in the PHB, qualified information, difficulties and obstacles, financial cost, challenges, childbirth safety, strengthening obstetric nursing, public policy of the PHB. And, in this final phase, in other words: 3) treatment of the results, interference and interpretation, they became significant and valid with the presentation of the categories, constituting a type of controlled interpretation, which could be based on the constitutive elements—meaning and code and by the sender and the receiver [20].

This stage was followed by a count of imperative codes, combined with the inductive themes that emerged in many of the interviews. Then there was the saturation of these codes, which occurred through the recurrence of meanings, in which no new codes were discovered, only those already designated, thus expressing the realization of a dictionary of codes. These allowed for the design of the group of codes and the related citations and assimilation; thus, the units of acceptances, with the classification of constructive information and the regrouping of meanings, based on the non-primordial categorization [20] that emerged from the conjunction of the feedback from the sharers, which motivated the construction of the categories. The discussion was based on public policies in the field of labor and birth and the scientific literature on planned home births.

### Ethical considerations

The study followed the rules of the Declaration of Helsinki of 2013, guaranteeing the confidentiality and anonymity of all participants' data, and was approved by the Research Ethics Committee of the Institute of Health Sciences of the Federal University of Pará (CEP-ICS/UFPA), according to protocol no. 4.463.291/2020. To preserve confidentiality, anonymity and reliability, the interviewees were identified by: the letter (HP) of the health professional, followed by a numerical number, corresponding to the sequence of interviews (HP1, HP2, HP3, …, HP20), in addition to the guarantee of voluntary participation, by signing the Free and Informed Consent Form, through Google form.

## Results

As for the characterization of the participants, all were female, with 11 aged between 30 and 40, followed by eight aged between 40 and 50 and one interviewee aged between 20 and 30. Regarding marital status, ten were married, followed by single women with four participants, three in a stable union and three divorced.

The predominant ethnicity/race was white with nine participants, followed by brown with eight, indigenous with two and black with one. As for religion, seven had no religion, followed by Catholicism with six participants, Spiritism with three, Protestants with two and Afro-Brazilian religions with two.

The length of professional experience in obstetrics prevailed, with ten participants having more than ten years' experience, followed by eight with between five and ten years and two participants with between one- and five-years’ experience. Regarding the length of time, they had worked in providing care for planned home births: eight participants said they had between one- and five-years’ experience, followed by eight participants with between five- and ten-years’ experience and four participants who said they had more than ten years' experience.

Based on non-primordial categorization, the following categories emerged: 1) The home as a break from the obstetric model; 2) Challenges for planned home births in the Brazilian obstetric health panorama.

### The home as a break from the obstetric model

Given the prevailing obstetric scenario, based on the biomedical and technocratic model, obstetric care at home is a counterpoint to hospital care, which is medicalized and rigid. Home care helps based on scientific evidence, the creation of a bond, and as a humanized model with respect and a successful birth, according to the testimonies:

So, home birth nurses try to study the physiology of childbirth a lot so that you can effectively respect this body. And, obviously, to base their practice on scientific evidence so that you can be sure that you are offering the best possible care to the public. So that's basically it, you look for safety and evidence to be able to respect the physiology of the woman's body and, in this way, provide that physiology […] we favor that physiology to occur. Therefore, it's this respect for the environment, noise reduction, light reduction […] so we try to favor it, sometimes even bringing in something related to aromatherapy, chromotherapy, things that will improve that environment to favor physiology (HP1).

I think they first try to feel more welcome and comfortable at home than in a hospital environment, so most of the women who opt for home birth don't feel comfortable in a hospital environment […] I think they shy away from interventions, they know that at home they won't have an unnecessary intervention because we don't do them (HP19).

Home birth care imposes the need to establish guidelines and protocols that direct the performance of PHB care in a homogeneous way, aggregating the work and direction of the multi-professional team throughout the country, such as the absence of a regulation/resolution for obstetric nurses in Brazil:

So, we know that in the national context, each team has this assistance in one way, and we don't know how these teams prepare themselves because in principle, as there is no regulation, any professional who is there fit to attend a birth in a hospital environment can attend a home birth (HP4).

I believe that the professional practice of childbirth care is still not uniform because people are not uniform either. Each team is set up differently. So, we can't say that care is offered in this way because we don't know. There are teams that get to know each other and so on, as if they were working in an institutionalized way, with stricter criteria, only in the home model (HP5).

Qualified information is a facilitator of access to home births. For professionals, there is a link between access to an informed choice and deciding to have a PHB, as the testimonies show:

And part of it, too, is access to information, which most women don't know is a possibility, that it's safe to choose to have your baby at home. Good quality information. Data that gives women the security to understand and choose the location. Where the baby will be born. (HP3).

Because often the woman who accesses it has obtained enough information to feel safe, but this information is not available to everyone. It's available to women with a higher level of education. She needs to understand scientific study, to be able to read a scientific article and understand it. Perhaps she needs to be able to understand more technical language (HP20).

Health professionals, as individuals inherent in this cultural context, also construct points of view about home birth care that offer resistance and assume a stigmatizing, marginalized and hostile imaginary towards women who opt for home birth:

A class council is never going to open space for the obstetric nurse to work freely. And it's not always the council of this profession, sometimes it's their own colleagues too. And they say that we're crazy, that we're mad. They say it's going to go wrong (HP5).

Look, unfortunately I still see a lot of prejudice, both for the medical profession, and I'll tell you something else, even sometimes from nursing itself. Many nurses turn up their noses when you say you work in home births, they say you're crazy, that you're irresponsible. That childbirth must be in a hospital environment, things like that (HP16).

PHB care, in the professionals' perception, is a model that provides positive maternal and neonatal outcomes, based on a care practice that prioritizes natural childbirth, without interventions, that respects the care offered and is based on scientific evidence.

### Challenges for planned home birth in the Brazilian obstetric health panorama

The speeches revealed that the advance of technology has created a sense of security and protection, associating better maternal and neonatal outcomes with hospital births from a cultural perspective. Home birth, on the other hand, is perceived as a step backwards, a denial of modern medicine, and suffers significant resistance from society, as can be seen in the following accounts:

I believe that this favors the imaginary that home birth is something dangerous. Something that would put the life of the woman and the child at risk, when we know that it isn't, but I believe that the Brazilian population's national view of this event is still hampered by a lack of public policy, a lack of interest in this biomedical, hegemonic model, and that childbirth belongs to the institution and the doctor exclusively (HP4).

It was also observed that financial costs, linked to social issues, are an obstacle to women's access to home births:

Access, for women, is what ends up becoming an expensive service. As far as we know, most women can't afford it (…) we know that in the Brazilian reality it's something that's only for a few. Sometimes, the person doesn't even have the right to housing, the right to food, let alone the basic structure needed for a home birth (HP2).

And the other difficulty is that the model today is paid for. The model is paid for. So, if most women don't have the financial means to pay for this care, that's another limitation (HP7).

The work process in home birth care has gaps, such as difficulties for health professionals to act properly, such as the difficulty of acquiring specific materials and medicines for care, in other words, the infrastructure needed to guarantee care with the resources for care; in addition to the complete discontinuity of care when transferring the woman to a hospital institution, due to the lack of regulations for obstetric nurses to work in PHB in the country:

I see, for example, that to have a care bag with emergency medication, and even protocol medication such as oxytocin, I must do it in the immediate postpartum period, but I can't buy it. We must get a group together and find a supplier. If I need to use misoprostol, how do I do it? It's on COFEN's list, how do you do it? What if I need to use it at home until she can be transferred? Is that a problem? (HP15).

There is a need to strengthen obstetric nursing as the protagonist of this care through constant training and theoretical and practical updating based on current scientific evidence, which guarantees qualified access to Brazilian women in the process of giving birth. Thus, the topic should also be introduced during academic training, giving these professionals the tools to act in the face of possible complications and to deconstruct myths:

My opinion, you know, is that we should invest in, as you say, always training and improving, even training. I think urgency and emergency should exist in ABENFO and in COREN itself. That you have access to this qualification to be trained to identify urgency and emergencies (HP17).

I think that to attend home births, nurses would have to have a card, be authorized by COREN, have at least five years' experience in obstetrics, and take obstetric and neonatal emergency courses every year (HP18).

The speeches highlighted the urgent need for PHB to be included as equal access in the Brazilian Unified Health System, as there is no support or funding coverage from the public sector for this home care, which would universalize and democratize access for the population in general:

There's no way the SUS can offer something that isn't an established policy in the country. So, I believe that the policy would be the first point, to discuss this policy initially, to implement it so that we could, in private, follow up with it, with its backing (HP6).

Given this scenario, there is a stigmatization of the practice of PHB, which seems to make it difficult to take the necessary emergency measures when there is a need to transfer to a hospital:

Whenever this woman is transferred to a hospital, she is mistreated in this way, both by the woman and the whole team. And there's the whole issue of mistreatment and obstetric violence at such a delicate time. And when she mentions that she was coming from a planned home birth, it's totally, it seems to change the scene of care, it's totally contrary to what it should be (HP11).

In this way, the PHB still has many gaps to be addressed, such as enabling access to all Brazilian women, to break down the challenges and promote care that values women and their needs.

## Discussion

Contrary to what medical organizations in Brazil have said, PHB care is a safe environment, supported by scientific evidence, and countries such as the Netherlands, Sweden and the United Kingdom recommend home births for low-risk women. What is happening is a growing attempt to establish an increased risk of PHB, a fact not proven in the specialized literature, but rather of the hospital environment, which has greater negative outcomes in labor and birth, as well as an increase in unnecessary obstetric interventions, which is why the WHO does not recommend its use [21].

Then, with the emergence of maternity hospitals, the birthing process began to involve routines in hospital environments, where women's individuality was no longer considered; and, with this institutionalization of labor, procedures such as episiotomy, trichotomy, enema, and indiscriminate induction of labor began to be adopted, without these practices having been previously evaluated by scientific evidence [22].

Health professionals working in the home setting provide care that is a counterpoint to the rigidity of hospital protocols, promoting individualized and less interventionist care with a focus on innovation for the humanization of labor and birth. Home birth is a way of avoiding interventions and restrictions associated with the hospital, including delivery in the supine position, continuous monitoring of the fetal heartbeat and the indiscriminate use of instrumental delivery [22]. The PHB constitutes a break with the technocratic and biomedical model of institutionalized childbirth in a hospital environment for a birth focused on humanization, with the centrality of the woman with her self-knowledge of her body and herself, in a mutual connection with her birth ritual.

The ambience of the home represents a welcoming and safe atmosphere for women in labor, reinforced by the attitude adopted by health professionals who respect the physiology of labor and the autonomy and empowerment of women. Giving birth in a familiar environment provides greater freedom of movement and the ability to be surrounded by supportive and trustworthy people, giving a sense of comfort, control and freedom that would not be possible in a hospital environment [23]. Thus, respecting the physiology of birth as well as providing a comfortable and safe environment leads to a more successful birth.

In countries with well-integrated obstetric care, the perinatal outcomes for planned home births are not statistically different from planned hospital births [23]. In these countries, there is a lack of homogeneity in home care, revealing a diversity of approaches and specificities in the formation of teams [23]. Thus, a wide-ranging change in care is needed to offer homogeneous care based on scientific evidence.

The specialized literature establishes the importance of using protocols that standardize procedures, as they help health professionals make decisions; they make it possible to correct non-conformities, allow all workers to provide standardized care, as well as providing greater satisfaction for the patient and safety for the entire multi-professional team [24]. These facts reveal the need to establish guidelines and protocols that standardize PHB care by the country's health regulatory bodies, such as the Ministry of Health; the professional councils; and the Nursing and Medical Councils, with the aim of stimulating good practices, giving due direction to the multi-professional teams throughout Brazil.

Political decisions on the accessibility of home births depend on safety issues, but also on the financial viability of the family making this choice [25]. When families opt for PDP, they consider safety, but also the cost of local delivery. Corroborating this fact, the challenges faced by professionals in promoting home births are related to the difficulty of access for families due to financial reasons, as well as access to qualified information.

Studies carried out in Spain [25–27], reinforce these factors as limiting access for the entire population, since the Spanish public system does not offer this modality and there is no support for alternative birthing centers to hospitals. This forces the couple to intentionally seek information and pay the full cost of the service.

In Brazil, both the Public Health System and supplementary health plans do not offer coverage for PHB for women and their partners, who must mobilize their own financial resources to defend their right to choose the place of birth that seems most appropriate to them. In line with national studies, where most women had higher education [28–30], our study revealed that women's level of education gives them greater access to information and knowledge, encouraging them to question current models of care, as well as to seek out trained and qualified professionals. This shows the elitist nature of home birth care in the country, demonstrating the lack of PHB care for women with low economic conditions and vulnerability.

The lack of public policies on this issue results in a lack of guidelines to guide health professionals, and is reflected in society, which has almost no information about this possibility of choosing a place of birth. In this way, the Brazilian Ministry of Health does not have a network that supports the real needs of women in the process of pregnancy and childbirth. PHB care belongs only to those with greater economic power, generating an elitization of services and social exclusion. Thus, there is a gap in the quality of information related to the PHB care model, leading to the dissemination of low-quality information, both by health professionals and by society in general. And the subject assumes a stigmatizing, marginalized and hostile position in the social imaginary towards those who opt for or provide home care [30].

This prejudice is based on the curative paradigm, disseminated for decades by health professionals themselves and resonated by society, who consider home birth to be an inappropriate practice and who understand technology as synonymous with quality and safety. This is how the obstetric scenario is shaping up, where the practice of home birth rescues natural childbirth with little or no use of technological devices, as opposed to technical and interventionist care. The idea of PHB has become socially established as a step backwards, as it denies women the benefits of progress in modern medicine [26]. Thus, the human right of women prevails in this break from the obstetric model to home birth.

It is worth noting that this is a legal and recognized practice, although not encouraged by the Brazilian Ministry of Health. The Brazilian Guideline for Normal Childbirth Care reaffirms the recommendations on home birth proposed by the public body of the UK Department of Health, concluding, through studies, that low-risk normal childbirth is also safe in an out-of-hospital environment; and that there are no differences in the outcomes of births attended by obstetric nurses or midwives, clarifying that low-risk childbirth care carried out by these professionals is safe [21].

Still in relation to the legality and marginalization of the practice, the National Women's Health Commission of the Federal Nursing Council published technical opinion No. 3/2019. The commission takes a favorable stance on the legality, safety and freedom of obstetric nurses and midwives to perform low-risk deliveries in this environment [31].

However, there is a difficulty in accessing this right both for the Federal Nursing Council, which does not have any regulations or ordinances that legitimize the work of obstetric nurses in PHB, and a public health policy in the country, factors that prevent access for Brazilian women. There is also the medical profession, which considers home birth a risk and is totally opposed to this modality and the participation of medical professionals in care; through Recommendation 01/2012, it considers normal childbirth a potentially risky event and, due to the availability of technology in hospitals, recommends this location as safer and a priority for childbirth [32].

Given this scenario, PHB care is stigmatized, especially the violence experienced by women and home care teams in a hospital environment when a transfer occurs. Behaviors and attitudes of retaliation, blaming and hostile treatment for the professionals who receive these people in the health unit are described.

Thus, the specialized literature shows that, when receiving a woman who has attempted a home birth, some health professionals stigmatize her for adhering to a practice that is not accepted by the professional's opinion. In an American study of 2,700 women, the significant role of the place of birth was observed. It was found that women who needed to be transferred to the hospital from a planned home birth or from a normal birth center as an emergency in response to emergent complications experienced high rates of mistreatment and abuse [33].

This occurrence characterizes a violation of human rights, so it is important to guarantee timely transfer linked to hospital reception free of judgments and mistreatment. In 2014, the WHO issued a declaration on the prevention and elimination of disrespect during childbirth, encouraging health professionals to consider meeting the sociocultural, emotional, and psychological needs of women to avoid possible mistreatment and abuse [32].

Some authors characterize this multifactorial phenomenon as a socio-political mechanism that keeps the multi-professional collaboration fragmented at the time of transfer, such as lack of an ethical stance for hospital teams; concern for professionals about going against the recommendations of their own professional bodies; lack of information about the work process of obstetric nurses at home; invisibility, for the country's health policies, about this work model; and lack of effective communication between care teams and health services [24–34].

This establishes a scenario that fosters the social construction of hospital birth as the only safe place, and that giving birth at home is associated with a lack of health care. However, the current home birth care model aims to legitimize the role of obstetric nurses and midwives in conducting normal, low-risk births, transferring the acquisition of the equipment and supplies needed for the care process for women and their newborns to the responsibility of these professionals [35].

In this sense, there are gaps in the work process due to the professional's actions, such as the difficulty in acquiring specific materials and medicines for care, the lack of logistical support and even the complete discontinuation of their care when transferring the woman to a hospital. Regardless of the need to transfer to a hospital, the PHB professional needs support from the health system to obtain medicines that are restricted to hospital institutions, and the difficulty in acquiring them is an obstacle to providing PHB care.

It is important to note that the American College of Obstetricians and Gynecologists has listed criteria for safe care, including a high degree of integration of home care professionals with the obstetric system; professional training that meets the standards of the International Confederation of Midwives; ready access to prenatal consultations and facilitated intrapartum transfers, as well as the appropriate selection of pregnant women eligible for such a scenario [23]. It is therefore considered that the findings of this research show this limitation in the care offered. By reducing the degree of autonomy of obstetric nurses in home care, it is difficult to achieve better outcomes, factors that are essential for the safe performance of obstetric nurses in home birth care, revealing a weakness in the work process of these professionals.

Regarding this reality, strategies to strengthen obstetric nursing are essential to guaranteeing professional autonomy, including constant training, theoretical and practical updating based on current scientific evidence and the inclusion of the subject during academic training, factors which aim to ensure that nurses can perform to their full potential.

The exercise of professional autonomy is enhanced by the appropriation of theoretical knowledge, enabling resolute action in complex situations and the ability to make decisions. It is therefore essential that professionals who work in planned home births are always trained in current scientific evidence, since, according to scientific findings, obstetric nursing training alone does not prepare professionals for PHB [23, 36].

It is also necessary to broaden the discussion in professional training, especially in postgraduate courses, considering the international protocols for home birth care, which should then be better explored. Furthermore, it also reflects the need to change the basis of professional training, which is commonly centered on the biomedical and technocratic model, where there is no approach to the subject of planned home births, to prevent the perpetuation of stigmas and non-recommended care practices [37].

It is understood that these factors promote safe and qualified care, and it is important to point out that these professionals have legal backing and are qualified to provide low-risk childbirth care in a home environment. These skills are crucial to guaranteeing safe maternal and fetal health care, and the previous technical experience of the professionals involved in this type of childbirth care and their training to work in urgent/emergencies is essential, as is the constant theoretical and practical updating of these professionals [38].

Corroborating this, a study carried out in Northern Ireland found that home birth services are provided by midwives in collaboration with obstetricians to ensure that women receive care from a multidisciplinary team. However, most professionals reported feeling little or no support from their obstetrician colleagues in providing this care. Linking a multi-professional team to home care is essential for improving the good outcomes of this care, as well as indirectly breaking down paradigms about this care practice [39].

And the lack of medical support is often overcome by establishing an informal partnership with some professionals who favor the practice of humanized childbirth. Some said that they had expanded their professional network through their own experience, but also that often there was complete opposition to this practice and there was no possibility of a formal partnership within the team.

Diante do exposto, torna-se necessária a inserção do PDP como Política Pública no país, apontando como um equívoco o fato do Ministério da Saúde reconhecer o direito de escolha da mulher sobre o local de parto, os órgãos regulamentadores autorizarem a atuação das enfermeiras obstetras em domicílio, e ainda assim, não existir uma estrutura de trabalho desenhada pelas políticas de saúde que torne viável o atendimento ao parto domiciliar pelas enfermeiras obstétricas, pois não há amparo ou cobertura do financiamento por parte setor público para essa assistência que universalize e democratize o acesso para população em geral [40].

Considering the above, it is necessary to establish PHB as a public policy in the country. Although the Ministry of Health recognizes a woman's right to choose her place of delivery, and the regulatory bodies authorize obstetric nurses to work at home, there is no work structure designed by health policies to make it feasible for obstetric nurses to attend home births. This is seen as a mistake, as there is no support or funding coverage from the public sector for this type of care, which would universalize and democratize access for the general population [40].

It is therefore necessary for the Ministry of Health to establish a scenario for discussing planned home births and for the right to choose the place of birth to be guaranteed and made possible as part of women's sexual, reproductive, and human rights. It should also be included in the actions of the Brazilian Unified Health System, in the sense of a counter-hegemonic movement for change, requiring greater publicity about its potential.

A limitation of this study is the number of obstetric nurses who underwent the PHB, which was an obstacle to broadening the possible participants and contributing to a discussion about the object of study.

## Conclusion

This study aimed to understand obstetric nurses' perceptions of planned home birth care in Brazil. It showed a scenario of care practices based on scientific evidence, with a woman-centered approach, where there is respect for the preferences and wishes of the women in labor, providing qualified information and promoting her autonomy during the childbirth process.

In this sense, the Ministry of Health does not recognize the scientific evidence of home birth in Brazil, which does not constitute a public policy for access and care for Brazilian women. There is also a lack of regulations and resolutions from the Federal Nursing Council regarding the role of obstetric nurses in PHB, which legitimizes and provides institutional support for their professional practice.

There is a need to establish protocols and guidelines that standardize care practice at home to ensure greater safety, quality, and equity in care, as well as regulating the practice of home birth, either by the Ministry of Health or the Federal Nursing Council. By establishing guidelines based on scientific evidence and adapted to the Brazilian reality, it is possible to define eligibility criteria for pregnant women for planned home births, as well as to establish appropriate training standards for health professionals, to guarantee clinical competence and the ability to make resolute decisions.

The difficulty in accessing planned home births due to financial issues and the lack of qualified information is a major challenge still faced in the country, revealing the need for public policies that encourage the dissemination of evidence-based information and guarantee equitable access to this birth option. For as long as the Ministry of Health, as the body responsible for formulating health policies, does not encourage and recommend PHB, this reality will not change. This condition limits women's choice and changes the care model.

It is essential to implement new policies that cover the entire core of professionals' activities, as well as guaranteeing access to the population. Policies aimed at regulating subsidies, facilitating financial access to services and supplies for planned home births. By working to overcome these barriers, public institutions are contributing to more woman-centered childbirth care and their needs, providing a successful birth experience.

## Data Availability

The data sets used and/or analyzed during this study are available and under the domain of the corresponding author upon plausible justification and/or referred to in the informed consent (Diego Pereira, diego.pereira.rodrigues@gmail.com).
